# Self-compassion influences PTSD symptoms in the process of change in trauma-focused cognitive-behavioral therapies: a study of within-person processes

**DOI:** 10.3389/fpsyg.2015.01273

**Published:** 2015-08-27

**Authors:** Asle Hoffart, Tuva Øktedalen, Tomas F. Langkaas

**Affiliations:** ^1^Research Institute, Modum BadVikersund, Norway; ^2^Department of Psychology, University of OsloOslo, Norway

**Keywords:** self-compassion, posttraumatic stress disorder, prolonged exposure, imagery re-scripting, within-person processes

## Abstract

Although self-compassion is considered a promising change agent in the treatment of posttraumatic stress disorder (PTSD), no studies of this hypothesis exist. This study examined the within-person relationship of self-compassion components (self-kindness, common humanity, mindfulness, self-judgment, isolation, over-identification) and subsequent PTSD symptoms over the course of therapy.

**Method:** PTSD patients (*n* = 65) were randomized to either *standard prolonged exposure*, which includes *imaginal exposure (IE)* to the traumatic memory, or *modified prolonged exposure*, where *imagery re-scripting (IR)* of the memory replaced IE as the imagery component of prolonged exposure in a 10 weeks residential program. They were assessed weekly on self-compassion and PTSD symptom measures. The centering method of detrending was used to separate the variance related to the within-person process of change over the course of treatment from between-person variance.

**Results:** The self-compassion components self-kindness, self-judgment, isolation, and over-identification had a within-person effect on subsequent PTSD symptoms. These relationships were independent of therapy form. The within-person relationship between self-judgment and subsequent PTSD symptoms was stronger in patients with higher initial self-judgment. By contrast, there were few indications that within-person variations in PTSD symptoms predict subsequent self-compassion components.

**Conclusion:** The results support the role of self-compassion components in maintaining PTSD and imply the recommendation to facilitate decrease of self-judgment, isolation, and over-identification and increase of self-kindness in the treatment of PTSD patients. The reduction of self-judgment appears to be most important, especially for patients with a high initial level of self-judgment.

## Introduction

Some trauma-exposed individuals develop posttraumatic stress disorder (PTSD) as a consequence of the traumatic experience. PTSD individuals are intensely preoccupied with current threat from external (e.g., be attacked again) as well as internal (e.g., self-criticism) sources (Ehlers and Clark, 2000). Indeed, self-criticism and associated shame may be central maintaining factors in many cases of PTSD (Cox et al., 2004; Matos and Pinto-Gouveia, 2010; Øktedalen et al., 2014). As compassion from others and self-compassion is the most important factor in the adaptive regulation of emotional reactions to threat (Gilbert, 2000), developing self-compassion could be particularly helpful for PTSD individuals. This development should be especially relevant when the trauma survivor is facing the internal threat of self-criticism (Gilbert, 2000). If he/she can be kind, understanding and mindful toward the self when things are difficult, then the resulting response is likely to be one of feeling cared for and understood. This can be contrasted to the feelings of shame, defeat and submission elicited by self-criticism and attack.

According to Neff’s (2003) initial formulation, self-compassion entails three bipolar components: (a) self-kindness – being kind and understanding toward oneself in instances of pain or failure versus being harshly self-critical, (b) common humanity – perceiving one’s experiences as part of the larger human experience versus seeing them as separating and isolating, and (c) mindfulness – holding painful thoughts and feelings in balanced awareness versus over-identifying with them. However, empirical analysis by use of the Self-Compassion Scale (SCS; Neff, 2003) indicated that the six poles rather represented six separate but correlated factors. Thus, for instance self-judgment and self-kindness are not mutually exclusive, so that having low levels of one behavior do not necessarily mean having high levels of the other. As a result, Neff proposed a six-factor structure with one higher-order factor of self-compassion.

A few studies have examined the relationship between self-compassion and PTSD. Thompson and Waltz (2008) administered the SCS to students and among those who had experienced a trauma, SCS correlated negatively with PTSD avoidance symptoms. Harman and Lee (2010) suggested that lack of self-compassion may be accentuated in shame-based PTSD and found that high levels of self-critical thinking and low levels of self-reassuring thinking were associated with high levels of shame within a PTSD sample. They concluded that it might be an inability to develop self-kindness and self-reassurance, as much as self-criticism, that may contribute to the maintenance of PTSD.

Self-compassion has not been a central target in trauma-focused cognitive-behavioral therapies, which are the ones that so far have been documented to be efficacious for PTSD (NICE, 2005). For instance, prolonged exposure (PE; Foa et al., 2007), which is the most extensively documented one, consists of imaginal exposure (IE) to the traumatic memory, repeated listening to tapes of the imagery sessions, and *in vivo* exposure to avoided situations and stimuli. PE focuses more on the reduction of fear and other trauma-related negative emotions than on fostering self-compassion. However, in a modified version of the imagery component of PE – imagery re-scripting (IR; Smucker et al., 1996) – the fostering of self-compassion is one of the goals. The re-scripting involves that the patient’s Current Self – after an initial phase of reliving the traumatic memory in imagery – is invited to enter the imagery at the worst moment of the trauma, bring the situation to a solution (e.g., overpower a perpetrator), and then interact with the Traumatized Self back-then. The Current Self-Traumatized Self interaction may stimulate the development of self-compassion instead of shame, guilt, and self-critique. In an open trial, IR was found extremely helpful for PTSD patients who had previously not profited from standard PE (using IE as the imagery component; Grunert et al., 2007). In pilot studies, both loving-kindness meditation and mindfulness based stress reduction have shown promising results for veterans with PTSD (Kearney et al., 2013, 2014).

Understanding the role of self-compassion in the process of therapeutic change depends on the method used to examine it. Therapy process research has mainly focused between-patient data, that is, how differences in process among patients are related to differences in outcome among the patients. However, this level of analysis partly misses its target because psychotherapy theories and therapists focus primarily on *within-patient processes of change* without the confounding influence of variance related to individual differences. For instance, a therapist is interested in whether a successful facilitation of self-compassion in a patient at a certain point in therapy may lead to subsequent reduction of that patient’s symptoms. Only repeated measures data allow for the proper disaggregation of between-person and within-person effects (Curran and Bauer, 2011). When a set of measures is collected at a single point of time from multiple individuals, the resulting data provide information only about between-person relationships. In contrast, when a set of measures are collected at multiple points in time from multiple individuals, the resulting data contain information about both between-person and within-person differences. Such data must be carefully specified to avoid confounding the two sources of variability. A disaggregation of the between-person and within-person variance components of a predictor not only allows the study of within-person processes separated from between-person effects, but also is able to examine cross-level interactions of between- and within-person effects. For instance, the effect of experiencing more self-compassion than expected for a particular patient may matter more for patients who have lower self-compassion in general. When the general (between-person) level of self-compassion is low, for example when the patient usually exhibits no self-kindness and longs for this, the occurrence of some self-kindness in a particular session might be a valued event with an immediate effect on symptoms. On the other hand, when the patient’s self-kindness is high already and not an issue for him/her, the same increase would probably have less consequence.

The main purpose of the present study was to examine the role of self-compassion in the process of change from week to week during therapy in patients diagnosed with PTSD. The patients were randomly assigned to receive either *standard PE*, which includes IE, or *modified PE*, where IR replaced IE as the imagery component of PE in a 10 weeks residential program. They were assessed repeatedly (weekly) on self-compassion and PTSD symptom measures, allowing us to separate the variance related to individual differences (between-person component) at the start of treatment from variance related to the intra-individual process of change during treatment (within-person component). The outcome of the two treatments is reported elsewhere, showing few differences between them at post-treatment. The uncontrolled effect sizes (Hedges’ g; Borenstein et al., 2009) on the PTSD Symptom Scale-Interview (PSS-I; Foa et al., 1993) at post-treatment was 1.31 (95% CI: 0.80–1.83) and for standard PE, and 0.84 (95% CI: 0.47–1.21) for modified PE. Effects for both treatments were significantly higher than the literature control benchmark (*Z* = 3.29, *p* < 0.001 for standard PE and *Z* = 2.14, *p* < 0.05 for modified PE) indicating that that both treatment implementations were effective as compared to untreated controls. The effect of modified PE was significantly lower than the PE literature benchmark (*Z* = -2.01, *p* < 0.05), whereas the effect of standard PE was not (*Z* = -0.25, n.s.), indicating that the performance of standard PE was close to results reported for PE in the treatment literature, whereas the one of modified PE was not (unpublished data). Based on the considerations above, we wanted to examine the following hypotheses:

(1) Self-compassion will increase over the course of therapy.

(2) Time-specific change in a patient’s self-compassion over the course of therapy will be negatively related to subsequent change in PTSD symptoms assessed 3 days later (within-person effect). That is, when self-compassion for a given patient is higher than is expected for that patient, subsequent symptoms will be lower.

(3) There will be a cross-level interaction of between-person and within-person effects. That is, the less self-compassion at the start of therapy, the stronger the relationship between time-specific change in self-compassion and subsequent change in PTSD symptoms will be during therapy, and the more self-compassion at the start of therapy, the weaker the relationship between time-specific change in self-compassion and subsequent change in PTSD symptoms will be during therapy.

Related to Harman and Lee’s (2010) proposal that the lack of self-kindness may be as important as self-criticism in contributing to PTSD symptoms, we tested the hypotheses above for each of the positive and negative components of self-compassion as formulated by Neff (2003). To explore the possibility of a reversed relationship between self-compassion and PTSD symptoms, we examined whether time-specific change in a patient’s PTSD symptoms over the course of therapy will be negatively related to subsequent change in self-compassion assessed 4 days later (within-person effect). Finally, due to the explicit focus on self-compassion in IR in contrast to IE, we explored whether patients receiving IR would change more in self-compassion over the course of treatment than those receiving IE, and whether the within-person relationship between self-compassion and PTSD symptoms would be stronger in IR within PE than in IE within PE.

## Materials and Methods

### Participants

The participants were selected from referrals to a PTSD treatment program at a National clinic. The clinic has been established for specialized residential treatment of non-psychotic patients who lack adequate local treatment opportunities or have not responded adequately to outpatient care. The study eligibility was similar to treatment eligibility, that is, all patients who were considered to potentially benefit from the PTSD treatment were included. The inclusion criteria were: (a) satisfying Diagnostic and Statistical Manual of Mental Disorders (fourth Edn; DSM-IV; American Psychiatric Association [APA], 1994) criteria for PTSD, (b) PTSD identified as the primary disorder in need of treatment, (c) age 18–67 years, and (d) accepting withdrawal of all psychotropic medication (regulated by the hospital – patients referred to the hospital have usually received medication without effect). The exclusion criteria were: (a) extensive dissociative symptoms, (b) suicidal risk, (c) current psychosis, (d) current active alcohol/substance abuse, and (e) ongoing trauma (e.g., current involvement in an abusive relationship). The study was approved by the Regional Ethic Committee and the patients’ gave informed consent after the procedures had been fully explained.

**FIGURE 1:**
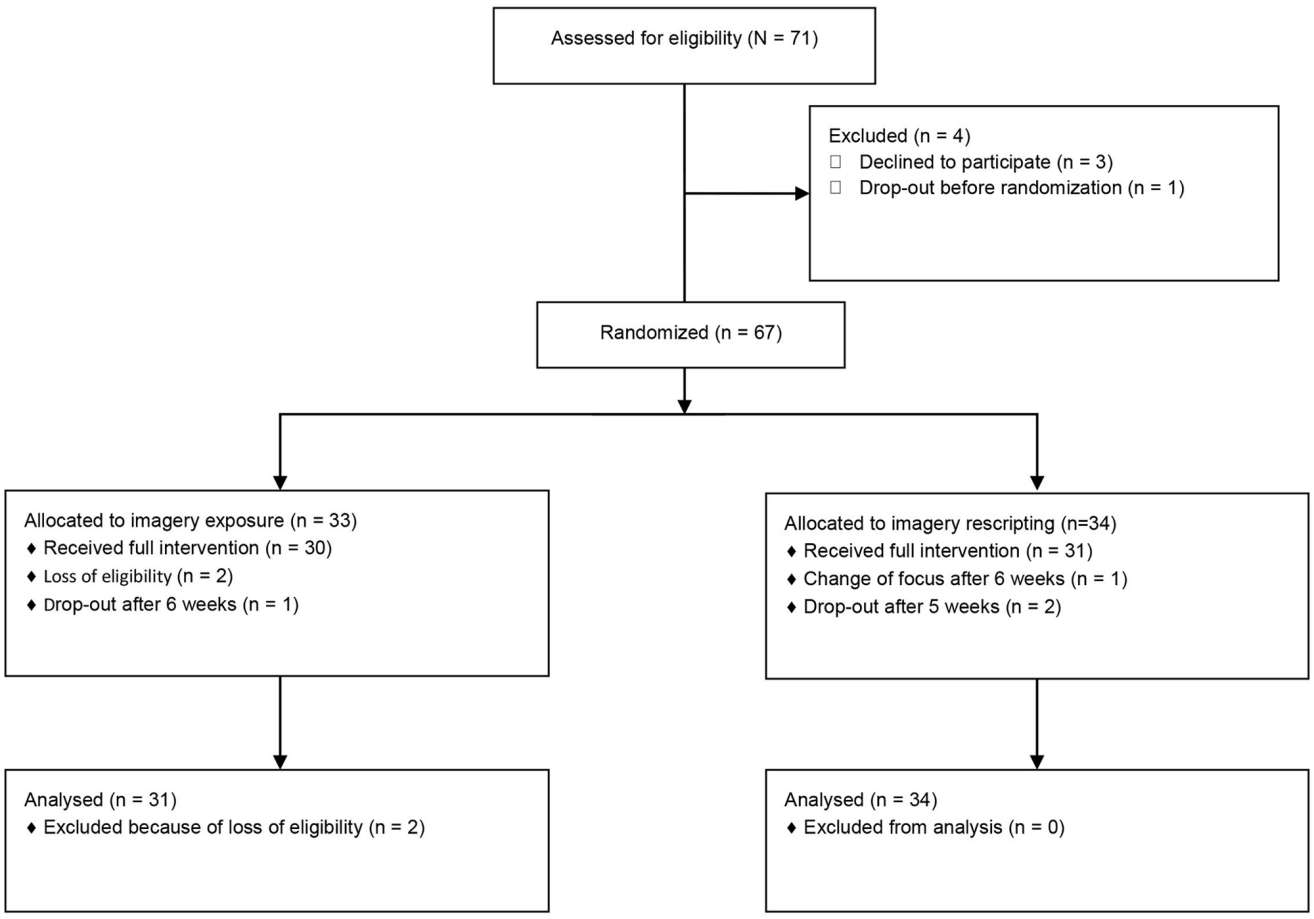
**Flowchart of patients included in the study**.

A flow chart of patients is presented in **Figure 1**. Seventy-one patients were found eligible for treatment at an assessment stay and admitted to treatment from December 2008 to November 2010. As shown in **Figure 1**, we ended up with an intent-to-treat (ITT) with imagery sample consisting of 65 patients – 31 IE and 34 IR patients – who signed consent, were randomized to an imagery condition, and were not removed by the investigators. Of these, three patients dropped out within 5–6 weeks into the program.

The mean age of 65 ITT patients – 38 women and 27 men – was 45.2 years (SD = 9.7 years). The mean length of time since the index trauma was 17.5 years (SD = 13.3 years). Fifty-one (78%) reported having received previous treatment from a mental health professional, and 36 (55%) used psychotropic medication for PTSD symptoms prior to being admitted to treatment. Twenty-four (37%) reported a history of one or more suicide attempts.

The most prevalent index trauma, defined as the one experienced by the patient as currently most distressing or most frequently re-experienced or both, among the 38 women was non-sexual assault by a familiar person (*n* = 12, 31.6%), sexual assault by a familiar person (*n* = 9, 23.7%), and sexual assault by a stranger (*n* = 8, 21.1%). Among the 27 men, war experience was most frequent (*n* = 7, 25.9%), followed by assault by familiar persons (*n* = 6, 22.2%), and accidents (*n* = 4, 14.8%). Over half the index traumas were prolonged (a threatening situation not resolved within 1 day) and/or repeated events. The patients had a high level of Axis I and II co-morbidity. Most prevalent were anxiety disorders (*n* = 54, 83%) mood disorders (*n* = 50, 77%), and Cluster C personality disorders (*n* = 21, 32%).

### Process Measures

The PTSD Symptom Scale-Self-Report (PSS-SR; Foa et al., 1993) consists of 17 items corresponding to the DSM-IV PTSD symptoms. It overlaps considerably with the Impact of Event Scale – Revised (Weiss and Marmar, 1997), a widely used measure which also purports to measure DSM-IV PTSD symptoms. The PSS-SR is usually rated for the last week, but the rating period was shortened to the last 3 days in this study. The frequency part of the criteria was changed correspondingly (0 = not at all, 1 = 1 time/sometimes, 2 = 2 times/half of the time, 3 = 3 or more times/almost always). Symptom severity is determined by the sum of the 17 ratings. PSS-SR symptom severity has demonstrated satisfactory psychometric properties (Foa et al., 1993). It was translated to Norwegian by the first and the third author and back-translated to English by a native English speaking professional also competent in Norwegian, until satisfactory formulations were found. Internal consistency reliability of the pre-treatment PSS-SR rating was 0.88.

In the SCS, (Neff, 2003), subjects are instructed to indicate how often they act – in difficult times – in the manner stated in each of 26 items on a scale of 1 (almost never) to 5 (always). Subscale scores for self-kindness (five items), self-judgment (five items), common humanity (four items), isolation (four items), mindfulness (four items), and over-identification (four items) components of self-compassion are computed by averaging across items within each subscale. The SCS has demonstrated satisfactory psychometric properties (Neff, 2003). It was translated to Norwegian according to the same procedure as for the PSS-SR (see above). In our study, the mean internal consistency reliabilities of the SCS subscales across assessments were satisfactory for self-kindness (α = 0.90), common humanity (α = 0.78), self-judgment (α = 0.83), and isolation (α = 0.79), but low for mindfulness (α = 0.61) and over-identification (α = 0.60).

### Procedure

Random sequences generated from www.random.org were used for random assignment. A researcher independent of the study conducted the assignment procedure about a week after treatment had begun, as both treatment protocols were identical before session 3. In order to control for therapist effects, a blocked randomization procedure was used to ensure that each therapist was assigned an equal number of cases in each condition. The MINI International Neuropsychiatric Interview (MINI; Sheehan et al., 1994) for assessing DSM-IV Axis I disorders and the Structured Clinical Interview for DSM-IV Axis-II Personality Disorders (SCID-II; First et al., 1994) for assessing Axis II disorders were conducted at pre-treatment. The PTSD Symptom Scale-Interview (PSS-I; Foa et al., 1993) was used as primary outcome measure and conducted at pre-and post-treatment with high inter-rater reliability (unpublished data). All the interviews were conducted by independent interviewers blind to treatment allocation. The SCS (and other process measures) was completed weekly, that is, every Friday morning. The patients were asked to base their ratings on their experiences during the last 4 days, that is, during the most treatment-intensive part of the week. The PSS-SR was completed every Monday morning. The patients were asked to base their ratings on their experiences during the last 3 days, that is, during a less treatment-intensive period.

### Treatment

The outpatient manuals for PE (including IE; Foa et al., 2007) and IR (Smucker et al., 1996) were used, but adapted for the inpatient setting. Essentially, it meant that milieu therapists were available to assist in between-session assignments and to provide safety and support after intensive individual sessions. The patients received 10 individual sessions lasting 90 min over a period of 10 weeks. The first two individual sessions were the same for all patients and consisted of giving a general treatment rationale and provide trauma education (first session) and introduce and plan *in vivo* exposure by constructing an exposure hierarchy (second session). Then, before the third session, patients were stratified by therapist and randomly allocated to either the IE or the IR condition, and followed the relevant protocols for the third (occurring toward the end of the 2nd week of treatment) to ninth session. In the 10th and final session, the content was again identical and consisted of imagery exposure to the total memory, a review of progress, and suggestions of continued practice. In the 6th week, the patients returned home to test their newly acquired skill in their natural environment. Between each session, patients were given assignments to work on every day, consisting of *in vivo* exposure assignments and listening to audio recordings of the last session. The dose and duration of all interventions were equivalent in both conditions. The patients received treatment in a ward with other anxiety patients and participated in the ward common program, consisting of one ward meeting and one physical exercise session per week.

*Imaginal exposure* consisted of reliving the traumatic event in imagination and recounting the memory in the present tense as detailed and vivid as possible. The memory was repeated if necessary to allow total reliving of 40–60 min. The entire memory was relived the first two or three sessions. Thereafter, reliving was focused on the currently most distressing parts of the memory. The imagery experiences were verbally processed in post-imagery dialogs, which included discussions of maladaptive thoughts associated with the traumatic memory.

*Imaginal exposure* consisted of three continuous phases. The first phase consisted of imagery re-living of traumatic event in order to activate the trauma memory and to identify the hot spot(s). In Phase 2, without pause in imagery, the memory was relived from the beginning, but now – at the identified hot spot – the patient was asked to imagine the Current Self enter the scene at the hot spot and bring the situation to a solution (overpower the perpetrators or update the Traumatized Self back-then with future information). Finally, in Phase 3, the patient was stimulated to imagine an interaction between the Current Self and the Traumatized Self back-then. Typically, the Current Self will begin to care for the Traumatized Self practically (e.g., wash him/her) and emotionally (e.g., hold or hug him/her). When Current Self experiences negative feelings toward the Traumatized Self and finds it difficult to nurture him/her, he/she is asked to move closer to, look into the eyes of, and talk directly to him/her to gain more access to his/her pain and needs. As in IE, the imagery was supposed to last 40–60 min and was processed in post-imagery dialogs.

### Therapists

Treatment was delivered by two experienced therapists trained in cognitive-behavioral therapy (CBT). One therapist was a female psychiatric nurse with a master’s degree; the other was a male clinical psychologist with a Ph.D. The clinical staff also consisted of four psychiatric nurses assisting the patients in treatment assignments between sessions. The staff had a minimum of 10 years experience of delivering CBT for anxiety disorders and all had received CBT training and certification provided by the Norwegian Association for Cognitive Therapy.

### Training and Supervision

All the staff received pre-study workshops and supervision by the experts Elizabeth Hembree in PE including IE and Mervin Smucker in IR during several pilot treatment groups. Throughout the study period, all of the individual sessions were videotaped, and each of the experts provided 90 min supervision sessions of taped imagery biweekly. In addition, the first author provided two 60 min supervision sessions per week to the milieu staff and individual therapists in a group format.

### Treatment Integrity

The Treatment Integrity Checklist (Foa et al., 1997) contains items describing the ingredients of PE. The imagery component was the only component intended to differ between the two treatment conditions. Thus, we rated the eight items of the PE Sessions 4–9, Section C: IE. A corresponding checklist for IR was constructed. The checklists allowed computation of an adherence rating as a proportion. An overall adequacy (competence) rating for the episode was given using a 1–5 scale with the anchor points *poor*, *mediocre*, *satisfactory*, *good*, and *excellent*. Ten cases from each treatment were chosen at random (using www.random.org) and the imagery part of session five was rated by experts (Hembree for IE and Smucker for IR). The first and the second author rated the same sessions independently in order to estimate interrater reliability. The Intra-class Correlation [ICC (3, 2); Shrout and Fleiss, 1979] was 0.69 in IE and 0.92 in IR for adherence and 0.93 in IE and 0.87 in IR for adequacy. The mean expert adherence rating was 0.75 (SD = 0.15) in IE and 0.80 (SD = 0.21) in IR. Mean adequacy rating was 2.78 (SD = 1.30) in IE, corresponding to a level a little below satisfactory, and 3.20 (SD = 1.32) in IR, corresponding to a level a little above satisfactory.

### Statistical Analysis

A main purpose of this study was to examine how within-person changes in self-compassion affected subsequent within-person changes in outcome from week to week during treatment. Such a focus on within-person processes necessitates a proper disaggregation of the within-person and between-person components of change in the time-varying predictor. The choice of method of disaggregating within-person and between-person effects in a time-varying predictor depends on its trajectory of change (Curran and Bauer, 2011).

To estimate these trajectories of change, we conducted series of mixed models using the six self-compassion scales (self-kindness, self-judgment, common humanity, isolation, mindfulness, and over-identification) and the PTSD symptom measure (PSS-SR) as dependent variables. The ITT sample was analyzed. The fit of nested models was compared by the likelihood ratio test, in which the difference in model -2 log likelihood (LL) values is divided by the difference in degrees of freedom of the models (Fitzmaurice et al., 2004). Maximum likelihood (ML) estimation was used to compare the models.

We started with a model with only a fixed intercept and no random effects and with a diagonal covariance structure for the residuals, added a random intercept, and, finally, a random effect of week in therapy. After the best random effects structure had been found in this way, we tested whether alternative residual covariance structure [e.g., AR(1), Toeplitz] than the diagonal could improve model fit. We then tested whether the inclusion of a fixed linear time term (week in therapy) and – in a second step – a fixed quadratic time term (week^2^) as independent variables improved model fit. These nested models were compared by the likelihood ratio test. For all the SCS scales and for the PSS-SR scale, a fixed and random intercept with a fixed and random effect of time turned out to be most appropriate model. An unstructured covariance structure was used for the random effects, allowing the intercept and slope to co-vary. In addition, a first order autoregressive [AR(1)] covariance structure of the residuals improved model fit compared to the diagonal structure.

Because the time-varying predictors were characterized by a linear trajectory of change over time, we utilized the statistical centering method of detrending to disaggregate the within- and between-person variability in them (Curran and Bauer, 2011). We created two new variables representing the within-person change and between-person differences for self-kindness, self-judgment, common humanity, isolation, mindfulness, over-identification, and PSS-SR scores, respectively (see the applied equations in the supplementary material). First, we created the within-person predictor by regressing the variables on time separately for each individual using ordinary least squares (OLS). The resulting within-person deviations over weeks in therapy represent the within-person components of the time-varying self-compassion and symptom measures. In this way, the within person deviations are conceptualized as the difference between a time-specific observation and the trend line for the variable (i.e., the expected value given a linear growth in the variable). We used the estimated differences on the time-varying predictors at the first measurement occasion (pre-treatment) to represent their between-person component.

To correct for the possibility of Type I error, a sequential rejective approach to the study hypotheses was applied (Holm, 1979). The most extreme *p*-level was compared to the alpha significance level of 0.05 divided by the number of tested hypotheses (18), yielding a level of 0.0028. Then the next most extreme *p*-level was compared to 0.05/17 = 0.0029, and so forth. Because all of the hypotheses were directional in nature, one-tailed tests were used. For the exploratory comparisons, a liberal *p*-level of 0.05 (two-tailed) was used. The magnitude of effects were computed by pseudo-*R*^2^ for the proportion reduction in each variance component using the variance estimated from a model with fewer parameters relative to the variance estimated from a model with more parameters (see the applied equations in the supplementary material; Snijder and Bosker, 1999). We used the program SPSS 21.0.

## Results

### Summary Statistics for the Weekly Outcome and Self-compassion Measures

Missing data in the ITT sample was 6.4% for PSS-SR scores, and from 8.4 to 9.0% for the SCS subscales. For the most part, these missing data were due to the drop-out from treatment. The mean between-person PSS-SR score at pre-treatment (estimated intercept) was 32.97 (SD = 7.93). At pre-treatment, mean between-person self-kindness score (estimated intercept) was 2.25 (SD = 0.91), common humanity score was 2.47 (SD = 0.77), and mindfulness score was 3.06 (SD = 0.70). Among the negative subscales, mean between-person self-judgment (estimated intercept) was 3.44 (SD = 1.01), isolation score was 3.46 (SD = 0.90), and over-identification score was 3.21 (SD = 0.69). The SD of the within-person SCS subscales scores ranged from 0.34 (mindfulness) to 0.46 (isolation). Considering absolute values, the lowest correlation between the estimated between-person SCS subscales scores was between mindfulness and self-judgment (*r* = -0.10) and the highest was between self-judgment and isolation (*r* = 0.65). The inter-correlations for the within-person SCS subscale scores over the course of treatment were negligible to moderate: the highest in terms of absolute values was 0.43 between isolation and over-identification and the lowest was -0.02 between mindfulness and self-judgment.

### Testing Hypotheses

In testing the first hypothesis, the weekly scores on the SCS subscales were used as dependent variables in mixed models with random intercept and slope and an AR(1) covariance structure for the residuals (see Statistical Analysis section). Time (week) and treatment (IR within PE vs. IE within PE) were used as predictors. In support of our first hypothesis, scores for self-kindness, β = 0.044, SE = 0.012, *t*(74.5) = 3.54, *p* < 0.0036 (one-tailed); mindfulness, β = 0.039, SE = 0.011, *t*(68.3) = 3.55, *p* < 0.0033 (one-tailed); self-judgment β = -0.040, SE = 0.015, *t*(70.2) = -2.69, *p* < 0.0046 (one-tailed); isolation β = -0.102, SE = 0.016, *t*(75.9) = -6.27, *p* < 0.0028 (one-tailed); and over-identification, β = -0.083, SE = 0.013, *t*(83.4) = -6.16, *p* < 0.0029 (one-tailed) displayed change over the course of treatment. Common humanity was the only subscale that did not exhibit change, β = 0.021, SE = 0.014, *t*(71.1) = 1.48, n.s. (one-tailed). In a second step, a time by treatment interaction was added to explore the possibility of different degrees of change in the two treatments. However, the time by treatment interactions were non-significant for all variables (all absolute *t*-values < 1.55).

In testing the second hypothesis about a negative within-person effect of self-compassion on subsequent symptoms, the weekly outcome measure – the PSS-SR – was used as dependent variable in mixed models with random intercept and slope and an AR(1) covariance structure for the residuals (see Statistical Analysis section). Time (week), treatment (IR within PE vs. IE within PE), and the within-person and between-person components of the six SCS subscales were used as predictors. Separate analyses were conducted for each SCS subscale. To establish a temporal sequence between predictor and outcome, within-person SCS scores at Fridays were lagged and thus related to the PSS-SR scores the following Monday (3 days later). Our hypothesis about was supported for four of the six SCS subscales. The result for self-kindness is provided in **Table 1**. It shows that a patient with a higher than usual score on self-kindness in a given week had a lower than usual score on PSS-SR assessed 3 days later, β = -1.122, SE = 0.443, *t*(412.4) = -2.53, *p* = 0.0110 (one-tailed, borderline significant). There were corresponding within-person effects of self-judgment, β = 1.789, SE = 0.427, *t*(412.7) = 4.19, *p* < 0.0031 (one-tailed); isolation, β = 1.196, SE = 0.389, *t*(406.7) = 3.07, *p* < 0.0039 (one-tailed); and over-identification, β = 1.242, SE = 0.412, *t*(410.8) = 3.02, *p* < 0.0042 (one-tailed); on PSS-SR scores. By contrast, the same analyses using common humanity and mindfulness as within- person predictors yielded no significant results. In order to explore their relative contributions, the four significant within-person predictors were included in the same model. Only self-judgment remained close to significant β = 1.208, SE = 0.494, *t*(410.0) = 2.43, *p* = 0.0080 (one-tailed). All other absolute *t*-values were below 1.40.

**Table 1:** Fixed effects estimates (top) and variance–covariance estimates (bottom) for models of the predictors of PTSD symptoms.

Parameter	Model 1	Model 2


**Fixed effects**		
Intercept	40.807^∗^ (2.907)	43.647^∗^ (5.005)
Week	-1.435^∗^ (0.157)	-1.482^∗^ (0.420)
Treatment: IR	-1.747 (2.126)	-6.184 (6.173)
Treatment: IE	0 (0)	0 (0)
WP self-kindness	-1.122^∗^ (0.443)	-1.209 (1.529)
BP self-kindness	-3.061^∗^ (1.186)	-4.303 (2.280)
Week × treatment: IR	-	0.792^∗^ (0.303)
Week × treatment: IE	-	0 (0)
Week × WP self-kindness	-	0.019 (0.165)
Week × BP self-kindness	-	-0.165 (0.173)
Treatment: IR × WP self-kindness	-	-0.027 (0.891)
Treatment: IE × WP self-kindness	-	0 (0)
Treatment: IR × BP self-kindness	-	1.820 (2.663)
Treatment: IE × BP self-kindness	-	0 (0)
WP self-kindness × BP self-kindness	-	0.005 (0.507)
**Random effects**		
Residual	17.929^∗^ (1.720)	17.933^∗^ (1.724)
AR(1) rho	0.210^∗^ (0.077)	0.211^∗^ (0.077)
Intercept	59.030^∗^ (12.701)	58.200^∗^ (12.534)
Week	1.219^∗^ (0.284)	1.067^∗^ (0.257)
Intercept × Week	0.861 (1.405)	1.027 (1.265)
-2 log likelihood (LL)	3340.376	3305.338

*SE are in parentheses. IR, imagery re-scripting; IE, imaginal exposure; WP, within-person; BP, between-person; AR(1), first order autoregressive.*

*^∗^p < 0.05 (two-tailed).*

Compared to a baseline model including only the random effects (intercept, time) and the fixed effect of time, residual variance was reduced with 3.4% and random intercept variance with 11.5% when within- and between-person self-kindness scores were added in the model. The corresponding percentages were 3.6 and 20.3 for self-judgment, 3.7 and 7.6 for isolation, and 4.6 and 4.1 for over-identification.

To examine our third hypothesis, the six interactions between our four predictors were added in a second set of models (see Model 2 in **Table 1** as one example). This hypothesis, stating that a within-person effect of self-compassion on PTSD symptoms would be stronger with lower initial levels of self-compassion, was supported only for the self-judgment subscale. That is, there was a positive cross-level between-person by within-person self-judgment effect, β = 1.243, SE = 0.467, *t*(392.7) = 2.66, *p* < 0.0050 (one-tailed).

To explore the possibility of reciprocal causation – that within-person changes in PTSD symptoms would be related to subsequent self-compassion – the six SCS subscales were used as dependent variable and the between- and within-person components of PSS-SR scores were used as predictors in mixed models. PSS-SR scores assessed on a Monday were related to SCS scores assessed the following Friday to assure a temporal precedence of the PSS-SR scores. The only within-person effect that appeared was a small one for over-identification, β = 0.011, SE = 0.005, *t*(454.8) = 2.10, *p* < 0.05 (two-tailed). Residual variance was reduced with 3.4% and random intercept variance with 9.6% when within- and between-person PSS-SR scores were added in the model.

### Treatment Differences in the Process of Change in PTSD

To explore the effect of treatment on the within-person relationship of self-compassion and PTSD, also the cross-level interactions of treatment and the within-person SCS predictors were included in the second set of models. **Table 1** shows that the cross-level interaction for self-kindness was not significant. That is, the within-person relationships between self-kindness and outcome did not differ in the two treatments. This interaction was non-significant also for the other five SCS subscales. It should be noted that there was a time by treatment effect, the PSS-SR scores were less reduced in IR than in IE (see **Table 1**).

## Discussion

The main purpose of this study was to examine the role of self-compassion components in the process of therapeutic change in PTSD patients. The first hypothesis, stating that self-compassion would increase over the course of therapy, was mainly supported by the results. Two of the three positive components of self-compassion – self-kindness and mindfulness – increased, and all the three negative components – self-judgment, isolation, and over-identification – decreased over the course of therapy. The positive component common humanity, viewing one’s suffering as part of the larger human experience, did not change over the course of therapy. The patients may have felt increasing common humanity with other trauma or PTSD sufferers, but this was not measured.

The second and most central hypothesis of a within-person effect of self-compassion on subsequent PTSD symptoms was supported for self-kindness and all the three negative components of self-compassion. Accordingly, when self-kindness for a given patient was higher than was expected for that patient, the subsequent symptom score was lower than was expected for him/her. Furthermore, when the self-judgment, isolation, or over-identification score for a given patient was lower than was expected for that patient, the subsequent symptom score was lower than was expected for him/her. The four significant time-varying predictors explained from 3.4 to 4.1% of the outcome variance. Accordingly, our results suggest that both the removal of factors that tend to block self-compassion – self-judgment, isolation, and over-identification – as well as fostering self-kindness lead to improvement in PTSD symptoms in the process of therapeutic change. However, the negative factors were most consistently found to be influential on the time-specific change in PTSD symptoms. Furthermore, when all the significant predictors were included in the same model to assess their relative contribution, only the negative factor self-judgment remained significant. The central position of self-judgment is consistent with other results from the present sample that within-person changes in shame and, to a lesser extent, guilt, predicted subsequent changes in PTSD symptoms (Øktedalen et al., 2014) as well as with several cross-sectional studies of the relationship between self-criticism/shame and PTSD symptoms (Cox et al., 2004; Harman and Lee, 2010; Matos and Pinto-Gouveia, 2010).

Thus, against Harman and Lees’ (2010) conjecture, the negative components of self-compassion turned out to be the most important ones for symptom change in the treatment of PTSD. A reduction of self-judgment, isolation, and over-identification may lead to a reduction of shame, guilt, and loneliness associated with the traumatic memory, thus relieving the potential for intrusive re-experiencing and avoidance of reminders. Similarly, an increase of self-kindness may counteract the negative emotions of the memory and therefore symptoms.

For the most part, within-person changes in PTSD symptoms did not predict subsequent self-compassion. That is, *time-specific change* in a person’s symptoms during therapy was not related to this person’s subsequent *change* in self-kindness, common humanity, mindfulness, self-judgment, or isolation. There was a small within-person effect of symptoms on over-identification. However, this exploratory result is in extra need of replication.

In our third hypothesis, we expected that self-compassion would be of greater concern for those who had a lower individual level and thus be more influential in these persons’ process of change. This hypothesis involving a cross-level interaction was supported only for the self-judgment subscale. Consequently, the within-person effect of self-judgment on subsequent PTSD symptoms was stronger in those with higher initial self-judgment. Given that self-judgment is related to shame, this supports that self-compassion processes are most important in patients with shame-related PTSD (Gilbert, 2000).

Even though one of the treatment conditions (IR within PE) contained a part focused on fostering self-compassion, while the other one (IE within PE) did not, the results did not support that this part was successful. Both the changes in the self-compassion components and the within-person relationships between these components and outcome were independent of the specific therapy form. The patients’ blocks to self-compassion, for instance fear of self-compassion (Gilbert et al., 2011), may have been too severe to be influenced by the Current Self-Traumatized Self interaction used in IR.

As the self-compassion changes appeared not to be a result of symptom changes, it seems that factors common to both treatment conditions were involved to increase self-kindness and reduce self-judgment, isolation, and over-identification. Trauma-related negative thoughts were addressed in post-imagery dialogs in both conditions and changes in negative thoughts about the self and the world have been shown to mediate changes in PTSD symptoms (Zalta et al., 2014). Indices of cognitions studied in Zalta et al. (2014; e.g., “I am inadequate”) may overlap with indices (items) of self-compassion on the SCS (e.g., “I am disapproving and judgmental about my own flaws and inadequacies,” self-judgment item). Thus, self-compassion change may reflect change in negative cognitions. Alternatively, self-compassion may have been elicited by viewing oneself from an observer perspective during the reliving of the trauma. It may also be speculated that a compassionate attitude toward the self by the reduction of self-judgment, isolation and over-identification and the increase of self-kindness are fostered indirectly in the therapeutic relationship and not directly in the IR experiences. For example the accepting stance of the therapist may lead to emotional corrective experiences and be internalized by the patient.

Self-compassion and PTSD symptoms were assessed weekly and adequate methods were utilized to separate the within-person and between-person effects of the time-varying predictors in the applied multilevel models. Thus, we could study within-person relationships over the course of therapy, which are of particular relevance for psychotherapy theories. This is because therapy theories concern how change in a process variable relates to subsequent change in an outcome variable during therapy. Such knowledge directly informs therapists what process variables need to be affected to achieve patient improvement. By contrast, knowledge of between-person relationships – one patient having low initial self-compassion and poor outcome and another having high initial self-compassion and good outcome – does not imply that an increase in the first patient’s self-compassion would lead to better outcome for that patient. That is, between-person findings cannot be used as evidence that working with a given patient to improve self-compassion will improve outcome for the same patient. Thus, relationships established on a between-person level do not imply that the same relationships hold on a within-person level.

A further advantage of properly separating the between- and within-person components of a time-varying predictor is the possibility to examine cross-level interactions of within- and between-person effects. For therapists, how between-person differences in for example self-compassion moderate within-person relationships over the course of therapy is more directly relevant than the correlations of these differences with overall outcome. Such moderating knowledge inform therapists under what conditions (e.g., high self-judgment relative to other patients) certain within-person change processes are working (e.g., higher than usual self-judgment on a given time-point predicts higher than usual PTSD symptoms). The disaggregation procedure renders the time-varying components completely uncorrelated to any time-invariant variable. Thus, we are left only with time-varying confounders, which there may be not so many of. Consequently, we can have increased confidence that the obtained relationships actually are causal. Actually, positive and negative components of self-compassion could influence each other (Neff, 2003) and thus act as confounders for each other, but our measurement schedule and time level were not adapted to studying the sequential relationships among these components.

A further advantage of studying within-person relationships in repeated measurement data is the possibility to identify reciprocal or even reversed causality between process and outcome. The RCT design, where patients were randomized to two empirically based imagery methods, allowed us to study the possible moderating influence of therapy form on the within-person relationships. The studied sample had high clinical representativeness as research eligibility was similar to treatment eligibility and only 3 (4.2%) of 71treatment eligible patients declined research participation. Moreover, the drop-out rate from imagery treatment was low, 3 (4.6%) of 65 ITT patients.

The studied patients had experienced a variety of traumas – often of a prolonged and/or repeated type – and all patients considered eligible for treatment were also considered eligible for the study. Such a general and clinically representative sample is suited for an initial investigation of self-compassion in PTSD treatment as all PTSD patients experience current threat and self-compassion should therefore be relevant for all of them.

The present study has several limitations. Although both treatments performed better than literature control benchmark, and standard PE (including IE as the imagery component) did not perform poorer than the literature benchmark for this therapy, the adequacy ratings were only around a level of satisfactory for both imagery treatments. Thus, the varied component of treatment may have been delivered in a less than optimal way. No integrity ratings were performed for the other components of treatment (e.g., *in vivo* exposure). In the present study, the internal consistency appeared satisfactory for four of the SCS subscales, but low for mindfulness and over-identification. Self-compassion and symptom ratings were collected from the same individual, that is, the patient, and this may inflate their correlation. However, halo effects were prevented by having the ratings done 3 and 4 days apart. Furthermore, response biases like acquiescence are supposed to cut across ratings and may affect within-person variations – which were the main focus of this study – to a lesser degree.

We studied process on a weekly time scale, and larger or lesser scales could be associated with different results. The strategy of using the same therapists across therapies has both strengths and weaknesses. The therapists may not be equally competent and have same preferences for both therapies. Actually, one of the therapists reported preference for IR (unpublished data). However, this bias could not explain the results as PTSD symptoms measured weekly were less reduced over the course of therapy in IR than in IE.

Conceptually, we have followed Neff’s definitions of self-compassion as a super-ordinate construct entailing six components throughout this report. It should be noted that the definition of the three positive components – self-kindness, common humanity, and mindfulness – does not fully overlap with the traditional definition of compassion as a sensitivity to the suffering of self and others with a desire to relieve it (Gilbert, 2000). Moreover, it should be considered to disconnect the so called negative components of self-compassion – self-judgment, isolation, and over-identification – from the self-compassion concept and view them as separate phenomena. A factor analysis of the SCS in a community sample did not support a six-factor solution with a higher order self-compassion factor, but suggested a two-factor solution, formed by the positively and negatively formulated items respectively (López et al., 2015). The separation of positive and negative components is further supported by a fMRI study of Longe et al. (2010) where very different neuronal substrates of self-reassurance and self-criticism were identified.

As elaborated above, the present study invites an increased focus on within-person relationships in psychotherapy research. The finding of clear within-person relationships between self-compassion (as defined by Neff) and symptoms of PTSD, despite a limited focus on self-compassion in the treatments, suggest that a lack of self-compassion may maintain PTSD and that treatments enhanced in components promoting self-compassion such as the mindful self-compassion program (Neff and Germer, 2013) should be examined. In particular, the existing pilot studies of mindfulness programs for PTSD (Kearney et al., 2013, 2014) should be followed up with more rigorous studies. Moreover, the studied PTSD sample in the present study was a severe one with high degree of co-morbidity, long duration of PTSD, and over half of the patients had experienced repeated and/or prolonged traumas. The resistances to self-compassion may have been particularly strong in this group (Gilbert et al., 2011). Future studies should assure that sufficient self-compassion training is provided and investigate the within-person relationships between self-compassion and outcome across therapies and type and severity of PTSD disorders. A different time scale than weekly should also be investigated. Furthermore, studies of within-person relationships between therapy events/therapist actions and self-compassion components are needed.

## Conclusion

The present within-person results make a firm basis for the recommendation to monitor and facilitate decrease of self-judgment, isolation, and over-identification and increase of self-kindness in the treatment of PTSD patients. The reduction of self-judgment appears to be most important, especially for patients with a high initial level of self-judgment.

## Author Contributions

AH has initiated and contributed to the conception and design of the study, has conducted the analyses, and has been the primary writer of the MS. TØ has contributed to the conception and design of the study, the collection of data, revised the MS. for intellectual content, and approved of the version to be published. TL has contributed to the conception and design of the study, the collection of data, revised the MS. for intellectual content, and approved of the version to be published.

## Conflict of Interest Statement

The authors declare that the research was conducted in the absence of any commercial or financial relationships that could be construed as a potential conflict of interest.
