# Supplementary material for: Self-compassion influences PTSD symptoms in the process of change in trauma-focused cognitive-behavioral therapies: a study of within-person processes
Source: Front Psychol. 2015 Aug 27;6:1273. doi: 10.3389/fpsyg.2015.01273 (PMC4551056; doi:10.3389/fpsyg.2015.01273)
Supplement: Supplementary file 1 [file Presentation_1.PDF]

Supplementary material

Equations for the multilevel models

Level 1: $y_{ti} = \beta_{0i} + \beta_{1i} x_{ti} + e_{ti}$ (1)

$e_{ti} = z_{ti} - \beta_{0i} - \beta_{1i} x_{ti}$

In the Level 1 model, individual-specific value of PTSD symptoms (y_{ti}) is a function of an individual intercept (β_{0i}), the slope coefficient of the time score for time t for individual i (β_{1i} x_{ti}) and the residual symptoms of PTDS (e_{ti}) on time t for individual i. The equation term e_{ti} is computed by deviating the time-specific predictor (z_{ti}) from the regression line ($\beta_{1i} x_{ti}$) estimated separately (case by case) for each individual in the sample. The deviated measure, e_{ti} , is then the residual (i.e., the observed score minus expected value) from the regression of the time-varying predictor on time computed separately for each individual case, which then represents the variable for within person level of each predictor (i.e., shame and guilt).

The Level-2 between-person predictor represents variance due to inter-individual differences in the time-varying predictor at start of treatment, as shown in Equation 2.

Level 2: $zb_i = \beta_{1i} x_{0i}$ (2)

In the Level-2 model, zb_i is the between-person component of the time-varying predictor and is a function of individual differences in the time-varying predictor at start of treatment (β_{1i} x_{0i}).

The equations for the model with main effects of the between-person and the within-person predictor are presented in Equation 3.

Level 1: $z_{ti} = \beta_{0i} + \beta_{1i} x_{ti} + e_{ti}$ (3)

$e_{ti} = (z_{ti} - \overline{z}_i) - (\gamma_{10} + u_{1i})(x_{ti} - \overline{x}_i)$

Level 2: $\beta_{0i} = \gamma_{00} + u_{0i}$

$\beta_{1i} = \gamma_{10} + u_{1i}$

$u_{0i} = (z_i - \gamma_{00}) - (\gamma_{10} + u_{1i})\,\overline{x}_i$

Composite: $E_i\,(z_{ti}) = (\gamma_{00} + u_{0i}) + \gamma_{10}E_i\,(x_{ti}) + E_i\,(u_{1i}\,x_{0i})$

$= (\gamma_{00} + u_{0i}) + (\gamma_{10} + u_{1i})\,E_i(x_{ti})$

In the Level-1 model, individual-specific value of symptoms of PTSD (y_{ti}) is a function of an individual intercept (β_{0i}), the within-person effects of the time-varying predictor ($\beta_{1i}\,x_{ti}$) and the residual PTSD symptoms (e_{ti}) on time t for individual i. In the Level-2 model, the individual intercept (β_{0i}) is a function of a fixed intercept (γ_{00}) and an individual-specific random intercept (u_{0i}). The individual effects of slope (β_{1i}) is a function of the fixed effects in rate of change (γ_{10}) and person specific slope (u_{1i}).

Equations for proportion reduction of error at each level

Proportion Reduction of Error for Predicting Level-1 Outcome:

$$R^2_{L1} = 1 - \left(\frac{residual\ variance\ more + intercept\ variance\ more}{residual\ variance\ fewer + intercept\ variance\ fewer}\right)$$

Proportion Reduction of Error for Predicting Level-2 Outcome:

$$R^2_{L2} = 1 - \left(\frac{\frac{residual\ variance\ more}{\#\ Level\ 1\ units} + intercept\ variance\ more}{\frac{residual\ variance\ fewer}{\#\ Level\ 1\ units} + intercept\ variance\ fewer}\right)$$
